# Patient-reported outcomes in meta-analyses – Part 1: assessing risk of bias and combining outcomes

**DOI:** 10.1186/1477-7525-11-109

**Published:** 2013-07-01

**Authors:** Bradley C Johnston, Donald L Patrick, Jason W Busse, Holger J Schünemann, Arnav Agarwal, Gordon H Guyatt

**Affiliations:** 1 Department of Anesthesia and Pain Medicine, and Institute of Health Policy, Management and Evaluation, University of Toronto, Toronto, ON, Canada; 2 Child Health Evaluative Sciences, Hospital for Sick Children Research Institute, Toronto, ON, Canada; 3 Department of Health Services, University of Washington, Seattle, WA, USA; 4 Seattle Quality of Life Group, Seattle, WA, USA; 5 Department of Clinical Epidemiology and Biostatistics, McMaster University, Hamilton, ON, Canada; 6 Department of Anesthesia, McMaster University, Hamilton, ON, Canada; 7 Department of Medicine, McMaster University, Hamilton, ON, Canada; 8 Faculty of Health Sciences, McMaster University, Hamilton, ON, Canada

**Keywords:** Patient-reported outcomes, Health-related quality of life, Meta-analysis, Systematic review, Health care decision-making

## Abstract

Systematic reviews and meta-analyses of randomized trials that include patient-reported outcomes (PROs) often provide crucial information for patients and clinicians facing challenging health care decisions. Based on emerging methods, guidance on combining PROs in meta-analysis is likely to enhance their usefulness.

The objectives of this paper are: i) to describe PROs and why they are important for health care decision-making, ii) illustrate the key risk of bias issues that systematic reviewers should consider and, iii) address outcome characteristics of PROs and provide guidance for combining outcomes.

We suggest a step-by-step approach to addressing issues of PROs in meta-analyses. Systematic reviewers should begin by asking themselves if trials have addressed all the important effects of treatment on patients’ quality of life. If the trials have addressed PROs, have investigators chosen the appropriate instruments? In particular, does evidence suggest the PROs used are valid and responsive, and is the review free of outcome reporting bias? Systematic reviewers must then decide how to categorize PROs and when to pool results.

## Introduction

Systematic reviews and meta-analyses of randomized control trials (RCTs) often include patient-reported outcomes (PROs). Including PROs is likely to be accompanied by issues of validity (can we trust the methods) and interpretability (what do the results mean), about which many systematic review authors are uncertain. The purpose of this article (Part 1) and a subsequent companion article (Part 2) is to familiarize systematic review authors with the nature of PROs and to provide guidance in negotiating the sometimes complex issues that they raise. Our discussion should be of interest to authors of systematic reviews and clinical practice guidelines, and other decision-makers wishing to take a critical perspective on how reviews have addressed issues of risk of bias and analysis of PROs. Much of the methodology laid out in this paper is also relevant to proxy-reported outcomes.

Clinical trials evaluating medical treatments and health interventions increasingly incorporate self-reported measures from patients, often referred to as PROs. According to the US Food & Drug Administration (FDA) Guidance for Industry - Patient-Reported Outcome Measures: a PRO is “any report of the status of a patient’s health condition that comes directly from the patient without interpretation of the patient’s response by a clinician or anyone else”. It can be measured in absolute terms (e.g., severity of a sign, symptom or state of a disease) or as a change from a previous measure [1].

## Why patient-reported outcomes?

**Figure 1:**
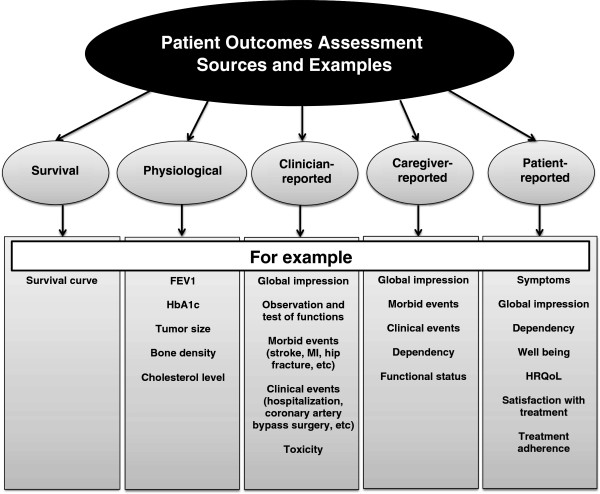
Sources and examples of patient outcomes.

PROs provide patients’ perspective on treatment benefit, directly measure treatment benefit beyond survival and major morbid events, and are often the outcomes of most significance to patients. Investigators sometimes choose PROs as primary outcomes; more often, PROs complement primary outcomes measured by survival, or major morbid events (e.g. stroke, myocardial infarction, disease exacerbation). Investigators also sometimes focus on biomarkers involving physiological, biological or laboratory-based measures (blood and tissue) or clinician-reported outcomes from various types of tests (biomarkers, physical examination), but these can only provide indirect evidence regarding patient-important outcomes [2]. Figure 1 provides examples of different outcomes that may be captured in clinical trials, including PROs.

Reports from patients may include sensations (most commonly classified as symptoms both of disease and treatment, sometimes referred to as side-effects), behaviours and abilities (most commonly classified as functional status), general perceptions or feelings of well-being, satisfaction with treatment, health-related quality of life (HRQoL), reports of adverse effects and adherence to treatment. PROs can be captured through interviews, self-completed questionnaires, diaries or other data collection tools such as hand-held devices and web-based forms. Although investigators may address these outcomes via proxy reports from caregivers, health professionals, or parents and guardians, these are not PROs.

Self-report measures often correlate poorly with physiologic measures. In asthma, Yohannes et al. found that variability in exercise capacity contributed to only 3% of the variability on a patient self-report questionnaire (the Breathing Problems Questionnaire; BPQ) [3]. In Chronic Obstructive Pulmonary Disease, the reported correlations between forced expiratory volume (FEV1) and HRQoL are relatively weak (r = 0.14 to 0.41) [4]. Similarly, in Peripheral Arterial Occlusive Disease, correlations between haemodynamic parameters and HRQoL were low [5,6]. In osteoarthritis, Hannan et al. showed discordance between radiographic arthritis and patient-reported pain [7]. These findings emphasize the limited value of surrogates for informing us about the impact of interventions on patient-important outcomes.

### PROs are key measures of treatment outcomes in some disease areas

PROs are most important when externally observable patient-important outcomes are unavailable, likely to be biased, or are rare. For many conditions, including pain syndromes, functional disorders, sexual dysfunction, emotional function and insomnia, PROs provide the only reasonable strategy for evaluating treatment impact.

Caregiver observed outcomes may be necessary in some conditions, such as advanced cancer and cognitive impairment, but PROs should be used whenever possible. The patient’s point of view should be of intrinsic interest to all stakeholders engaged in the area of health and illness. The ‘Checklist for describing and assessing PROs in clinical trials’ presents selected issues that authors should consider when reporting PROs in their reviews.

Checklist for describing and assessing PROs in clinical trials [8].

1. What were the PROs measuring?

1.1. What concepts or constructs were the PROs used in the study measuring?

1.2. What rationale (if any) for selection of concepts or constructs did the authors provide?

1.3. Were patients involved in the selection (e.g. focus groups, surveys) of PROs?

2. Omissions

2.1. Were there any important aspects of patient’s health (e.g., symptoms, function, perceptions) or quality of life (e.g. overall evaluation, satisfaction with life) that were not reported in this study?

3. If RCTs measured PROs, what were the instruments measurement strategies?

3.1. Did investigators use instruments that yield a single indicator or index number, a profile, or a battery of instruments?

3.2. If investigators measure PROs, did they use specific or generic measures, or both?

4. Did the instruments work in the way they were supposed to work – validity?

4.1. Was evidence of prior validation for use in the current population presented?

4.2. Were the instruments re-validated in this study?

5. Did the instruments work in the way they were supposed to work – ability to measure change?

5.1. Are the PROs able to detect change in patient status, even if those changes are small?

6. Can you make the magnitude of effect (if any) understandable to readers?

6.1. Can you provide an estimate of the difference in patients achieving a threshold of function or improvement, and the associated number needed to treat (NNT)?

*Based on Chapter 7 of Health Status and Health Policy, Guyatt et al, Users’ Guides to the Medical Literature: XII. How to Use Articles About Health-related quality of life.

## Description of PROs

Reviewers should understand the nature of the PROs used in each study, and communicate this information to the reader. Many different ways exist to label and classify patient outcomes, some of which are presented in Figure 1.

Health status and quality of life outcomes are an important category of PROs. Published papers often use the terms ‘quality of life’ (QoL), ‘health status’, ‘functional status’, ‘health-related quality of life’ (HRQoL) and ‘well-being’ loosely and interchangeably (see Table 1). For example, the meaning of QoL or HRQoL varies widely, ranging from psychosocial or patient-reported measures including those with limited evidence of validity, to well validated disease specific or generic HRQoL measures.

**Table 1:** Definitions of selected terms related to PROs

**Condition-specific Measure or Instrument**	A category of health measures that describes problems such as low-back pain or particular interventions or treatments such as knee-replacement or coronary artery bypass graft surgery.
**Disease-Specific Measure or Instrument**	A category of health measures of severity, symptoms, or functional limitations that are specific to a particular disease state, condition, or diagnostic grouping; for example, arthritis or diabetes.
**Domain** (also known as dimension)	PROs often have domains or dimensions as subcategories. For instance, the SF-36, a very popular instrument, has 8 domains or dimensions. Examples of domains defined for the SF-36 include: physical role functioning, social role functioning, emotional role functioning, and mental health. An alternative, less satisfactory designation is “subscale”.
**Functional Status**	An individual’s effective performance or ability to perform those roles, tasks, or activities that are valued, e.g. going to work, playing sports, or maintaining the house. Most often, functional status is divided into physical, emotional, mental, and social domains, although much finer distinctions are possible. Deviations from usual performance or ability indicate dysfunction.
**Generic Measure**	A measure designed for use with any illness groups or population samples, as opposed to those intended for specific illness groups.
**Health-Related Quality of Life**	Personal health status. It usually refers to aspects of our lives that are dominated or significantly influenced by our mental or physical well-being.
**Patient Satisfaction**	A consequence of the use of healthcare products, services or programs that affect patients’ satisfaction with health or healthcare.
**Quality of life**	An evaluation of all aspects of our lives, including, for example, where we live, how we live, and how we play. It encompasses such life factors as family circumstances, finances, housing and job satisfaction.
**Self-reported Symptoms**	Symptoms, which are directly reported by the patient by means of questionnaires, diaries, hand held devices or web-based forms.
**Well-Being**	Subjective bodily and emotional states; how an individual feels; a state of mind distinct from functioning that pertains to behaviours and activities.

The constructs captured in RCT outcomes can only be determined by examining the actual content of items or questions included in an instrument claiming to measure QoL or HRQoL. The labeling of concepts varies widely among researchers and few conventions apply. For example, an item measuring pain, a sensation known only to the patient, would be a symptom, or an aspect of QoL. Nonetheless, each item, subdomain, domain, or overall score addresses one or more concepts, which can be identified from the content (e.g. actual terminology used in the item).

Guidance from the GRADE working group is relevant to optimal approaches to using PROs in systematic reviews. GRADE is a system of rating confidence in estimates of effect (quality of evidence) that is extensively used and widely endorsed, including by the Cochrane Collaboration [9,10]. Using the GRADE process, the final product of a systematic review is an Evidence Profile or a Summary of Findings table (SoF) that presents, for each relevant comparison of alternative management strategies, the confidence in estimates for each outcome and, for dichotomous outcomes the best estimate of the magnitude of effect in relative terms and the absolute effect that one might see across sub-groups of patients with varying baseline or control group risks.

Currently, many primary studies do not seem to measure aspects of perceived health and QoL that are important to patients. A recent systematic review examining the type of outcomes selected and the prevalence of PROs in contemporary cardiovascular disease RCTs supports this notion: only 93 of 413 (23%) RCTs included patient-important outcomes as their primary measures. The study reported 122 of 174 (70%) RCTs where such outcomes would have been important or crucial excluded such outcomes, emphasizing the underuse of PROs with consideration to their relevance and importance to clinical decision-making [11]. One of the recommendations from GRADE and from the GRADE-associated Cochrane Applicability and Recommendations Methods group is that reviewers should begin the review process by defining and listing all patient-important outcomes [10,12] that are relevant to their question, which will include PROs. Patient-important outcomes often include morbidity, mortality, adverse events, hospitalization, function, disability, QoL, and inconvenience. Lacking evidence for important outcomes should be acknowledged rather than ignored to account for uncertainty surrounding reported results and clinical decision-making. This step is germane to the measurement of PROs. If primary studies fail to measure important aspects of patient perceptions, we may be much less confident regarding the treatment impact on PROs than we are about other outcomes. All patient-important outcomes should be included in a SoF table. In the extreme, there may be a line in the SoF table that is blank, because no study addressed this issue directly (and that blank line may refer to an important PRO). The careful prior consideration of all patient-important outcomes will highlight what is missing in outcomes reported in eligible RCTs.

If primary studies eligible for a systematic review have used PROs, it is worth considering the measurement strategies those PROs employed. Investigators may choose a single instrument that yields an overall score or indicator number (representing the impact of the intervention on mental or emotional function such as the Hospital Anxiety and Depression Scale), a health utility index number (again an overall score, but weighted in terms of anchors of death and full health), a profile (a series of scores, one for each dimension or domain), or a battery of tests (multiple PROs assessing different concepts or constructs) (Table 2).

**Table 2:** **A taxonomy of health status and quality of life measures**[13]

**Measure**	**Strengths**	**Weaknesses**
**Types of Scores Produced**		
Single **indicator** number	Global evaluation	May be difficult to interpret
	Useful for population	
Single **index** number	Represents net impact	Sometimes not possible to disaggregate contribution of domains to the overall score
	Useful for cost effectiveness	
**Profile** of interrelated scores	Single instrument	Length may be a problem
	Contribution of domains to overall score possible	May not have overall score
**Battery** of independent scores	Wide range of relevant outcomes possible	Cannot relate different outcomes to common measurement scale
		May need to adjust for multiple comparisons
		May need to identify the major outcome
Range of Populations and Concepts		
**Generic:** applied across diseases, conditions, populations, and concepts	Broadly applicable	May not be responsive to change
	Summarizes range of concepts	May not have focus of patient interest
	Detection of unanticipated effects possible	Length may be a problem
		Effects may be difficult to interpret
**Specific:** applied to individuals, diseases, conditions, populations, or concepts/domains	More acceptable to respondents	Cannot compare across conditions or populations
	May be more responsive to change	Cannot detect unanticipated effects
**Weighting System**		
**Utility:** preference weights from patients, providers, or community	Interval scale	May have difficulty obtaining weights
	Patient or consumer view incorporated	May not differ from statistical weights that are easier to obtain
**Equal-weighting:** items weighted equally or from frequency or responses	Self-weighting samples	May be influenced by prevalence
	More familiar techniques	Cannot incorporate tradeoffs
	Appears easier to use	

*Adapted from Patrick and Erickson, 1993.

If they have focused on HRQoL, trialists will have chosen generic or specific instruments, or a combination. If investigators were interested in going beyond the specific illness and possibly making comparisons between the impact of treatments on HRQoL across diseases or conditions, they may have chosen generic measures that cover all relevant areas of HRQoL (including, for example, self-care, and physical, emotional, and social function), and are designed for administration to people with any kind of underlying health problems (or no problem at all). These instruments are sometimes called health profiles; the most popular health profiles are short forms of the instruments used in the Medical Outcomes Study, such as SF-36 and SF-12 [14-16]. Alternatively (or in addition) RCTs may have relied on instruments that are specific to function (e.g. sleep or sexual function), a problem (e.g. pain), or a disease (e.g. heart failure, asthma, or cancer).

Another issue to consider is how the instruments are weighted. By convention, many specific instruments weight items equally because the scoring of multi-item scales is usually based on the average of component items. Utility instruments designed for economic analysis put greater emphasis on item weighting, attempting to ultimately present HRQoL as a continuum anchored between death and full health. Readers interested in a summary of these issues can look to an old, but still useful summary [17].

## Assessment of quality of evidence specific to PROs

**Figure 2:**
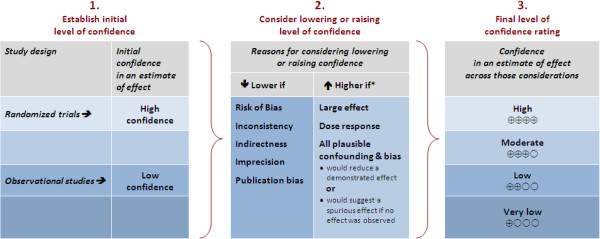
GRADE’s approach to rating quality of evidence (aka confidence in effect estimates).

Investigators use many instruments to capture PROs, and methods for developing, validating, and analyzing PRO data are diverse. In producing their SoF table, we suggest reviewers use the GRADE approach that identifies eight factors that influence confidence in an estimate of effect. While a body of evidence from RCTs starts at high quality, they may be assigned a lower rating because of risk of bias, imprecision, indirectness, inconsistency, or a high likelihood of publication bias. A body of evidence for an outcome from observational studies starts at low quality; factors that can increase confidence in estimates of effect are: large magnitude of effect, all plausible confounding would reduce the demonstrated effect (or increase the effect if no effect was observed) and a dose-response gradient. Figure 2 outlines the key factors for the assessment of the overall quality of evidence for a particular outcome.

Issues of particular relevance to PROs include problems in the validity of the instruments use (e.g., without extensive patient input, items and domains may not be both important to the target population and comprehensive with respect to patient concerns, and if not properly developed, instruments may not actually reflect the intended constructs), and issues of interpretability of findings (e.g. knowledge of the difference in score that represents small, medium, and large differences in HRQoL). Table 1 outlines the key issues in describing and assessing PROs.

### Validity

Validity has to do with whether the instrument is measuring what it is intended to measure. Content validity assessment involves patient and clinician evaluation of the importance and completeness of the content contained in the measures, usually obtained through qualitative research [18,19]. Construct validity is based on validation strategies developed by psychologists, who for many years have struggled with determining whether questionnaires assessing intelligence and attitudes really measure what is intended. Construct validity involves examining the logical relationships that should exist between assessment measures. For example, we would expect that patients with lower treadmill exercise capacity generally will have more dyspnea in daily life than those with higher exercise capacity, and we would expect to see substantial correlations between a new measure of emotional function and existing emotional function questionnaires. In rare cases, criterion validity may exist where there is a gold standard for self-report, usually a longer version of the instrument of interest.

When we are interested in evaluating change over time, we examine correlations of change scores. For example, patients who deteriorate in their treadmill exercise capacity should, in general, show increases in dyspnea, whereas those whose exercise capacity improves should experience less dyspnea; a new emotional function measure should show improvement in patients who improve on existing measures of emotional function. The technical term for this process is testing an instrument’s construct (or concurrent) validity.

Reviewers should look for evidence of the validity of PROs used in clinical studies. Unfortunately, reports of RCTs using PROs seldom review evidence of the validity of the instruments they use, but when available reviewers can gain some reassurance from statements (backed by citations) that the questionnaires have been previously validated.

A final concern about validity arises if the measurement instrument is used with a different population, or in a culturally and linguistically different environment than the one in which it was developed – typically, use of a non-English version of an English-language questionnaire. Ideally, one would have evidence of validity in the population enrolled in the RCT. PRO measures should, ideally, be re-validated in each study using whatever data are available for the validation: for instance, the relation between the PRO and other related outcomes measured. In the absence of empirical evidence of validity, reviewers are entitled to skepticism about the study’s PROs and may consider rating down the overall confidence in estimates on this basis [9].

### Responsiveness or ability to detect change

When we use instruments to evaluate treatment effects, they must be able to measure differences between groups, if differences do in fact exist. Randomization should ensure that patients in the intervention and control groups begin studies with the same status on whatever concept or construct the PRO is designed to measure. PROs must be able to distinguish among patients who remain the same, improve, or deteriorate over the course of the trial. This is sometimes referred to as responsiveness, sensitivity to change, or ability to detect change.

An instrument with a poor ability to measure change can result in false-negative results in which the intervention improves how patients feel, yet the instrument fails to detect the improvement. This problem may be particularly salient for generic questionnaires that have the advantage of covering all relevant areas of HRQoL, but the disadvantage of covering each area superficially [20].

In studies that show no difference in PROs between intervention and control, lack of instrument responsiveness is one possible reason. Suspicion about lack of ability to measure change is another potential reason for rating down the quality of evidence from a series of RCTs [9].

### Reporting bias

Studies focusing on PROs often use a number of instruments to measure the same, or similar constructs. This situation creates a risk of reporting bias. It is possible for investigators to measure a number of outcomes, and only report those that showed significant effects. Methodologists have long suspected the existence of outcome reporting bias [21,22], and systematic investigations comparing RCT protocols and their subsequent publications have provided estimates of its magnitude [23-25].

Investigators have examined a random sample of 156 completed Cochrane reviews that included 10 or more studies [26]. They found that a median of 46% of the review’s eligible trials (IQR: 20 to 75%, range: 2 to 100%) contributed to the pooled estimates. Thus, approximately half of the RCTs identified by the Cochrane reviews did not contribute to the pooled effect size in their meta-analyses. Furthermore, they found a correlation between effect size and the number of studies included (the fewer the studies, the larger the effect size) and this effect appeared strongest in studies using continuous outcomes (the correlation between the percentage of trials included in a meta-analysis and the SMD was -0.18 (95% CI: -0.35 to -0.01, p = 0.04). When analyses included less than 20% of eligible studies the mean effect size was 0.64 and when they included over 80% of the eligible studies the mean effect size was 0.31.

These results demonstrate just how frequently studies fail to provide data for meta-analyses, and provide support for the existence of reporting bias in which investigators are inclined to selectively report results with larger effects. Systematic reviews focusing on PROs should be alert to this problem. When only a small number of eligible studies have reported a particular outcome, particularly if it is a salient outcome that one would expect conscientious investigators to measure, reviewers should note the possibility of reporting bias and consider rating down confidence in estimates of effect in their summary of findings table [27].

## Outcome characteristics

### Deciding how to pool across studies

The definition of a particular PRO may vary between studies, and this may justify use of different instruments. Even if the definitions are similar (or if, as happens more commonly, the investigators do not define the PRO), the investigators may choose different instruments to measure the PRO. For example, the following instruments are all validated patient-reported pain instruments that an investigator may use in a primary study to assess an intervention’s usefulness for treating pain: the 20-item McGill Pain Questionnaire, the 7-item Integrated Pain Score, and the 56-item Brief Pain Inventory [28].

When deciding if statistical pooling is appropriate, reviewers will often find themselves reading between the lines to try and get a precise notion of the concepts or constructs underlying PROs. They may have to make at least a brief foray into the articles that describe the development and prior use of PRO instruments included in the primary studies. For example, authors of a Cochrane review of cognitive behavioural therapy (CBT) for tinnitus included QoL as an outcome [29], which was assessed in four trials using the Tinnitus Handicap Questionnaire, in one trial the Tinnitus Questionnaire, and in one trial the Tinnitus Reaction Questionnaire. The original sources are cited in the review. Information on the items and the concepts measured are contained in the cited articles, and review authors were able to compare the content of the instruments and conclude that statistical pooling was appropriate.

Systematic reviewers must decide how to categorize PROs and when to pool results. These decisions will be based on the characteristics of the PRO, which will need to be extracted and reported in the review. On most occasions, studies using PROs will make baseline and follow-up measurements and the outcome of interest will thus be the difference in change from baseline to follow-up between intervention and control groups. Ideally then, to pool data across two PROs that are conceptually related, one will have evidence of convincing longitudinal correlations of change in the two measures in individual patient data, and evidence of similar responsiveness of the instruments. Further supportive evidence could come from correlations of differences between treatment and control, or difference between before and after measurements, across studies. If one cannot find any of these data, one could fall back on cross-sectional correlations in individual patients at a point in time.

For example, the two major instruments used to measure HRQoL in patients with chronic obstructive disease are the Chronic Respiratory Questionnaire (CRQ) and the St. George’s Respiratory Questionnaire (SGRQ). Correlations between the two questionnaires in individual studies have varied from 0.3 to 0.6 in both cross-sectional (correlations at a point in time) and longitudinal (correlations of change) comparisons [30-32]. In a subsequent investigation, investigators examined the correlations between changes in the CRQ and SGRQ in 15 studies including 23 patient groups and found a correlation of 0.88 [33]. Despite this extremely strong correlation, the CRQ proved more responsive than the SGRQ: standardized response means of the CRQ (median 0.51, IQR 0.19-0.98) were significantly higher (p < 0.001) than those associated with the SGRQ (median 0.26, IQR -0.03-0.40). As a result, pooling results from trials using these two instruments could lead to underestimates of treatment effect in studies using the SGRQ [33-35].

Most of the time, unfortunately, detailed data such as those described in the previous paragraph will be unavailable. Investigators must then fall back on intuitive decisions about the extent to which different instruments are measuring the same underlying concept. For example, the authors of a meta-analysis of psychosocial interventions in the treatment of pre-menstrual syndrome faced a profusion of outcome measures, with 25 PROs reported in their nine eligible studies [36]. They dealt with this problem by having two experienced clinical researchers, knowledgeable to the study area and not otherwise involved in the review, independently examine each instrument - including all domains - and group 16 PROs into 6 discrete conceptual categories. Any discrepancies were resolved by discussion to achieve consensus. The pooled analysis of each category included between 2 to 6 studies. The ‘List of combinable instruments measuring similar constructs’ details the categories and the included instruments within each category.

List of combinable instruments measuring similar constructs:

Anxiety

Beck Anxiety Inventory

Menstrual Symptom Diary - Anxiety domain

State and Trait Anxiety Scale-State Anxiety domain

Behavioural Changes

Menstrual Distress Questionnaire-Behavioural Changes domain

Pre-Menstrual Assessment Form-Social Withdrawal domain

Depression

Beck Depression Inventory

Depression Adjective Checklist State-Depression domain

General Contentment Scale - Depression and Well-being domain

Menstrual Symptom Diary-Depression domain

Menstrual Distress Questionnaire-Negative Affect domain

Interference

Global Rating of Interference Daily Record of Menstrual Complaints – Interference domain

Sexual Relations

Martial Satisfaction Inventory-Sexual Dissatisfaction domain

Social Adjustment Scale - Sexual Relationship domain

Water Retention and Edema

Menstrual Distress Questionnaire-Water Retention domain

Menstrual Symptom Diary-Edema domain

## Summary

We have suggested a step-by-step approach to addressing issues of PROs in meta-analyses. This guidance on PROs and why they are important for health care decision-making, including the key risk of bias issues that reviewers should consider when combining PROs in meta-analysis is likely to enhance the usefulness of such overviews to end-users. In part 2 of this series, we will provide an overview of available methods for improving the interpretability of pooled estimates of PROs.

## Nomenclature

PROs, patient-reported outcomes

RCTs, randomized controlled trials

FDA, Food and Drug Administration

HRQoL, health-related quality of life

BPQ, Breathing Problems Questionnaire

FEV1, forced expiratory volume

QoL, quality of life

SoF, Summary of Findings

IQR, interquartile range

CI, confidence interval

SMD, standardized mean difference

CBT, cognitive behavioural therapy

CRQ, Chronic Respiratory Questionnaire

SGRQ, St. George's Respiratory Questionnaire

## Competing interests

The authors declare that they have no competing interests.

## Authors’ contributions

BCJ: concept, interpretation of data, manuscript drafting and preparation, administrative support, approval of final manuscript. DLP: concept, interpretation of data, manuscript preparation, approval of final manuscript. JWB: interpretation of data, manuscript preparation, approval of final manuscript. HJS: interpretation of data, manuscript preparation, approval of final manuscript. AA: manuscript preparation, critical appraisal and approval of final manuscript. GHG: concept, interpretation of data, manuscript preparation, approval of final manuscript. All authors read and approved the final manuscript.
